# Comment on “Clinical outcome of femoral neck system versus cannulated compression screws for fixation of femoral neck fracture in younger patients”

**DOI:** 10.1186/s13018-021-02709-7

**Published:** 2021-09-08

**Authors:** Yunfeng Tang, Guangling Wang

**Affiliations:** West China Hospital, Sichuan University, Chengdu, 610041 China

Dear Editor,

Recently, Hu et al. have published the article “Clinical outcome of femoral neck system versus cannulated compression screws for fixation of femoral neck fracture in younger patients” [1]. We read it with great interest. We would like to congratulate for their laudable efforts to perform this meticulous retrospective analysis on this intriguing subject. However, I would request the authors to further clarify upon certain points mentioned below so that the results could be more clearly interpreted and a similar study could be reproduced.

1. *Statistical analysis*. The authors mentioned “discrete variables were reported as numbers (percentage of total)”, but the statistical methods are not mentioned. A discrete variable is a variable whose value is obtained by counting. In contrast, continuous variable is a variable whose value is obtained by measuring [2]. Operation time, perioperative blood loss, and Harris score should be continuous variables, not discrete variables. The statistical analysis of discrete variables using continuous variables may eventually lead to bias.
2. *Study population.* In the study population, the authors mentioned that “with a minimum of 6 months follow-up” were included in the study. However, in the results, the authors also mentioned that “all patients received at least 1 year of clinical follow-up”; it seems to be a contradiction.
3. *Results*. In the results, surgical time of FNS and CCS was 79.75 ± 26.35 min vs. 64.58 ± 18.56 ml, which is incorrect. It should be 79.75 ± 26.35 min vs. 64.58 ± 18.56 min.

## Data Availability

All data generated or analyzed during this study are included in this published article.
